# Sleeve gastrectomy links the attenuation of diabetic kidney disease to the inhibition of renal tubular ferroptosis through down-regulating TGF-β1/Smad3 signaling pathway

**DOI:** 10.1007/s40618-023-02267-1

**Published:** 2024-03-21

**Authors:** C. Liu, M. Zhong, X. Jin, J. Zhu, Y. Cheng, L. Li, Q. Xu, Q. Liu, H. Ding, G. Zhang

**Affiliations:** 1 Department of General Surgery, Shandong Provincial Qianfoshan Hospital, Cheeloo College of Medicine, Shandong University, Jinan, 250014 China; 2 Department of General Surgery, The First Affiliated Hospital of Shandong First Medical University, No. 16766 Jingshi Road, Jinan, 250014 Shandong China

**Keywords:** Diabetic kidney disease, Sleeve gastrectomy, Ferroptosis, TGF-β1/Smad3 signaling pathway, Renal tubular injury, Bariatric surgery

## Abstract

**Purpose:**

To investigate how sleeve gastrectomy (SG), a typical operation of bariatric surgery, attenuated symptom, and progression of diabetic kidney disease (DKD).

**Methods:**

DKD model was induced by high-fat diet (HFD) combined with streptozocin in Wistar rats. SG was performed, and the group subjected to sham surgery served as control. The animals were euthanized 12 weeks after surgery, followed by sample collection for the subsequent experiment. The HK-2, a renal proximal tubular epithelial cell line derived from human, was utilized to investigate the potential mechanisms.

**Results:**

SG improved metabolic parameters and glucose homeostasis, and could alleviate DKD in terms of renal function indices as well as histological and morphological structures in DM rats, accompanied with a significant reduction in renal tubular injury. Compared with sham group, SG reduced the renal tubular ferroptosis. To further clarify the mechanism involved, in vitro experiments were performed. In the presence of high glucose, renal tubular TGF-β1 secretion was significantly increased in HK-2 cell line, which led to activation of ferroptosis through TGF-β1/Smad3 signaling pathway. Inhibition of TGF-β1 receptor and phosphorylation of Smad3 significantly ameliorated TGF-β1-mediated ferroptosis. In vivo experiments also found that SG improved the hyperglycemic environment, reduced renal TGF-β1 concentrations, and down-regulated the TGF-β1/Smad3 signaling pathway.

**Conclusions:**

With the capacity to lower the glucose, SG could attenuate the ferroptosis by inhibiting TGF-β1/Smad3 signaling pathway in DKD rats, and eventually attenuated DKD.

**Supplementary Information:**

The online version contains supplementary material available at 10.1007/s40618-023-02267-1.

## Introduction

Diabetic kidney disease (DKD), an insidious complication of diabetes mellitus (DM), is characterized by renal impairment or a glomerular filtration rate (GFR) < 60 ml/min/1.73 m^2^ and/or elevated urinary albumin excretion for at least 3 months [1]. Several factors have been reported to be responsible for the pathogenesis of DKD, especially the worsening insulin resistance (IR) independent of glycemic control [2]. Sleeve gastrectomy (SG), one of the typical operations of bariatric surgery, is an effective procedure for weight loss and glucose metabolism control, especially in those with type 2 DM [3]. Beyond weight loss, SG is reported to show many benefits, such as lowering hyperglycemia, hypertension, and hyperlipidemia [4]. Previously, we have focused on the roles of SG in ameliorating DKD symptoms and our data showed that SG could attenuate DKD in DM rats by upregulating the expression of nephrin and improving the structure of glomerular filtration membrane [5]. Since then, we tried to understand the potential mechanisms of how SG attenuated the symptom and delayed the progression of DKD.

In the past decades, most of the DKD studies have been focused on the role of glomeruli. Recently, the tubular hypothesis has been proposed in the nephron filtration and DKD. In fact, a change in GFR can be defined as a sum of tubular and vascular events, among which the tubular event is anything that directly affects tubular reabsorption and induces a change in GFR via tubuloglomerular feedback [1]. In the presence of simultaneous occurrence of vascular and tubular events, the tubular event is declared to be dominant. At the early stage of DKD, tubular impairment aggravates glomerular lesions. Thus, tubular injury is gradually becoming a predictor of DKD [1, 6]. On this basis, we hypothesized that renal tubular injury may serve as a bridge between SG and DKD.

Bariatric surgery could reduce apoptosis and alleviate oxidative stress in DKD rats [7]. Therefore, it is reasonable to speculate that bariatric surgery may ameliorate DKD through remission of cell deaths. As a new atypical form of cell death, ferroptosis provides a new explanation for the pathogenic mechanism of diseases with iron overload [8]. It contributes to disease progression, such as cancer and ischemic injury [9], as well as DM and its related complications [10]. To date, only a few studies proposed the link between ferroptosis and progression of DKD [11, 12]. Indeed, ferroptosis is involved in tubular cell death during the onset of acute kindy injury (AKI) [13]. Additionally, renal tubular cell impairment was significantly up-regulated in GPX4-null mice, which suggested ferroptosis may play important roles in renal tubular injury [14]. Thus, this study was designed to investigate the effects of SG on the renal tubular ferroptosis in the progression of DKD, and the potential mechanisms in this process.

## Materials and methods

### Animals

Male Wistar rats (4-week-old; 80–100 g), purchased from Weitong Lihua Experimental Animal Technology Co., Ltd. (Beijing, China), were housed in a specific pathogen free (SPF) animal room at Shandong Provincial Qianfoshan Hospital. For the adaptation, animals were initially fed with standard diet for 1 week and were free access to food and water in a regular light/dark cycle (12 h light: 12 h dark). The temperature was 22 ± 4 °C and the humidity was 50–60%. All experiment procedures were approved by Institutional Animal Care and Use Committee of Shandong Provincial Qianfoshan Hospital.

### Grouping

Rats were randomly divided into: (i) control (CON) group (*n* = 8), on a standard diet; (ii) DM group (*n* = 8), underwent DM induction according to the protocols of Animal Models of Diabetic Complications Consortium at week 9 [15]; (iii) SHAM group (*n* = 8) underwent DM induction followed by sham surgery at week 17; (iv) SG group (*n* = 8), underwent DM induction followed by SG at week 17. The flowchart of the grouping is shown in Supplementary Figure 1. The animals were euthanized at week 12 weeks after each treatment, followed by sample collection for the subsequent experiments. For the DM induction, rats were fed with a high-fat diet (HFD) purchased from Xietong Pharmaceutical Bioengineering (Nanjing, China) for 4 weeks, followed by intraperitoneal administration of streptozocin (STZ, 35 mg/kg, Sigma-Aldrich, St. Louis, MO, US) after fasting for 12 h. DM rats continued to receive HFD until euthanasia. About 8 weeks after STZ injection, the stable DKD rat model was established [16].

### SG procedures and sham surgery procedures

SG was performed at week 17 after fasting for 12 h according to the previous description [17]. Upon isoflurane-based anesthesia, a longitudinal incision (4 cm in length) was made under the xiphoid to expose the stomach. Later, the perigastric vessels were ligated using 7–0 thread (Chenghe Microsurgical Instruments Factory, Ningbo, China) and the greater curvature of the stomach was isolated. A 0.5 cm incision was made in the gastric fundus to empty the contents. Subsequently, the entire fundus, most of the gastric body, and part of the gastric sinus were resected along the longitudinal axis of the stomach, followed by anastomosis using a 5–0 thread. Animals in the SHAM group only received gastric exposure without the subsequent ligation of perigastric vessels and isolation of greater curvature [17].

### Fasting blood glucose (FBG), insulin concentration, and insulin resistance indices

Upon fasting for 12 h in each rat, blood was collected from tail vein at week 0, 3, 6, 9, and 12 after each treatment. Then, FBG was measured using a glucometer (Roche One Touch Ultra, Johnson & Johnson, CA, USA). Serum insulin level was measured at week 0, 3, 6, 9, and 12 after indicated treatment by EZRMI-13 K kit (Roche One Touch Ultra, Johnson & Johnson, CA, USA), according to the manufacturer’s instructions. HMOA-IR was calculated by multiplying fasting serum insulin and FBG, followed by dividing by the consistent 22.5, according to the previous description [18]. For the oral glucose tolerance test (OGTT), rats fasted for 12 h were administered with glucose (G8270, 1 g/kg, Sigma-Aldrich, St. Louis, MO, US) by gavage, followed by determination of the glucose concentration at 0, 15 min, 30 min, 60 min, and 120 min, respectively. The area under the curve (AUC) of the OGTT was calculated by the trapezoidal integration as previously described [19].

### Renal function evaluation

Renal function was evaluated based on measurement of serum and urine creatinine (Cr), serum urea, and urine microalbumin, respectively. Their concentrations were determined with commercial kits of Cr (Cat. No.: C011-2-1), urea (Cat. No.: C013-2-1), and microalbumin (Cat. No.: E038-1-1) purchased from Jiancheng Bioengineering Institute (Nanjing, China). Finally, the urinary albumin/Cr ratio (UACR) was calculated.

### Histological staining

Fresh renal tissues were fixed in 4% paraformaldehyde, and embedded in paraffin. The Sects. (5 μm) were subject to hematoxylin–eosin (HE) staining and periodic acid-Schiff (PAS) staining, which were used to measure the mesangial proliferation, glomerular basement membrane (GBM) thickness, and histologic structure of renal tubules.

### Transmission electron microscopy (TEM)

Fresh kidney cortices obtained from 12 week postoperatively were cut into fragments (1 mm × 1 mm × 1 mm), and were quickly immersed in fixative solution (Cat. No.: G1102, ServiceBio, Wuhan, China) for 6 h at 4 °C, followed by processing in 1% osmium tetroxide for 2 h at 4 °C. Subsequently, the samples were dehydrated through ethanol series, and were infiltrated using acetone and 812 embedding medium (905529-77-4, SPI). Upon polymerization, the sections were observed under TEM (HT-7700, Hitachi, Japan).

### TUNEL assay

TUNEL assay was conducted to determine the cellular death using paraffin section in the renal tissues, using commercial kit (Cat. No.: C10617, Thermo Fisher Scientific, USA). Cells in a state of death were labeled in green color, and were finally observed under a microscope at a wavelength of 488 nm.

### Immunohistochemistry (IHC)

For the IHC, sections were dewaxed by baking at 65 °C, hydrated, and antigenically repaired in a microwave oven using Citrate Repair Solution (Cat. No.: C1032, Solarbio, Beijing, China). The sections were incubated with primary antibodies including 4-HNE (Cat. No.: ab46545, 1:200, Abcam, Cambridge, UK), SLC7A11 (Cat. No.: 26864-1-AP, 1:200, ProteinTech, Wuhan, China), and GPX4 (Cat. No.: 67763–1-Ig, 1: 200, ProteinTech, Wuhan, China) overnight at 4 °C. Subsequently, the mixture was incubated with the goat-anti-rice and goat-anti-rabbit secondary bodies at 37 °C for 20 min. Finally, nuclear hematoxylin (Cat. No.: G1080, Solarbio, Beijing, China) staining was performed with commercial kit, according to the manufacturer’s instructions.

### Cell culture and treatments

HK-2, a proximal tubular epithelial cell line derived from human kidney, purchased from Procell (Wuhan, China) was utilized in this study. The cells were cultured in minimum essential medium (MEM) (Gibco, Carlsbad, CA, USA) containing 10% FBS (Gibco, Carlsbad, CA, USA) and 1% penicillin/streptomycin (Gibco, Carlsbad, CA, USA) at 37 °C in 5% CO_2_. Cell lines were identified by STR and were free of mycoplasma. Cells were first treated with MEM medium containing 10%FBS for 12 h. After a 6 h FBS-free starvation, subconfluent cells were collected for the subsequent grouping. Cells were divided into the following groups: (i) control group, cells cultured with MEM containing 1% FBS; (ii) TGF-β1 group, cultured with MEM containing 1% FBS and 10 ng/ml TGF-β1 (Cat. No.: ab50036, Abcam, Cambridge, UK) for 48 h; (iii) SB431542 group, cultured with MEM containing 1% FBS, 10 ng/ml TGF-β1 and 10 mM SB431542 (TGF-β1 receptor inhibitor) (Cat. No.:S1067, Selleck Chemicals, TA, USA); (iv) SIS3 HCl group, cultured with MEM containing 1% FBS, 10 ng/ml TGF-β1 and 10 mM SIS3 HCl (inhibitor of phosphorylation of Smad3) (Cat. No.:S7959, Selleck Chemicals, Houston, TA, USA); and (v) Fer-1 group, cells cultured with MEM medium containing 1% FBS, 10 ng/ml TGF-β1 and 100 mM Fer-1 (inhibitor of ferroptosis) (Cat. No.:S7243, Selleck Chemicals, Houston, Texas, USA).

### Cellular viability test

For each well of a 96-well plate, 5 × 10^3^ cells were seeded with 100 μL MEM medium containing 10% FBS and treated as mentioned above. Cell Counting Kit-8 (CK04, Dojindo Laboratories, Kumamoto, Japan) was utilized to determine the cellular viability, according to the manufacturer’s instructions. The wavelength was set at 450 nm.

### Lipid-ROS measurement

About 48 h after treatment, cells in each group were washed twice with PBS, and incubated with 5 mM C11-BODIPY (D3861, Thermo Fisher Scientific, USA) at 37 °C for 30 min. Afterward, the liquid in the plates was discarded and DAPI (C1002, Beyotime, Shanghai, China) was added to stain the nuclei, which were finally observed under a fluorescence microscope. Lipid-ROS fluoresces green under 488 nm excitation.

### Malondialdehyde (MDA), 4-HNE and glutathione (GSH) quantification

Expression of ferroptosis markers and ferroptosis-related chemical compound in tissues or cultured cells was measured using Lipid Peroxidation MDA Assay Kit (S0131S, Beyotime, Shanghai, China), 4-HNE ELISA Kit (E-EL-0128c, Elabscience Biotechnology Co., Ltd, Wuhan, China), and GSSG/GSH Quantification Kit (G263, Dojindo Laboratories, Kumamoto, Japan), respectively. All the tests were performed according to the manufacturer’s instructions. The results were colorimetrically quantified.

### Western blot

Total protein was extracted from renal cortex and cultured HK-2 cells with RIPA lysate containing 1% PMSF and 2% phosphatase inhibitor. The protein concentration was quantified with BCA method. Protein samples (50 μg of tissue protein; 20 μg of cellular protein) were separated on SDS-PAGE gels (PG212, EpiZyme, Shanghai, China). Then, the proteins were transferred to PVDF membranes, followed by blocking with 5% BSA (A8020, Solarbio, Beijing, China) for 1 h. Subsequently, the mixture was incubated overnight at 4 °C with primary antibodies, including 4-HNE (Cat. No.: ab46545, 1:1500, Abcam, Cambridge, UK), SLC3A2 (Cat. No.: 15193-1-AP, 1:5000, ProteinTech, Wuhan, China), SLC7A11 (Cat. No.: 26864-1-AP, 1:2000, ProteinTech, Wuhan, China), GPX4 (Cat. No.: 67763-1-Ig, 1:4000, ProteinTech, Wuhan, China), Smad2 (Cat. No.: 5339, 1:1000, Cell Signaling Technology, USA), p-Smad2 (Cat. No.: 18338, 1:500, Cell Signaling Technology, USA), Smad3 (Cat. No.: 9513, 1:1000, Cell Signaling Technology, USA), p-Smad3 (Cat. No.: 9520, 1:500, Cell Signaling Technology, USA), and Lamin B (Cat. No.: 12987-1-AP, 1:10000, ProteinTech, Wuhan, China). Afterward, the membranes were washed with PBST containing 2% Tween and incubated with goat-anti-mice and goat-anti-rabbit secondary antibodies for 1 h at room temperature. Finally, the protein bands were visualized with ECL (Millipore, USA) and quantified with ImageJ software (NIH, USA).

### Statistical analysis

SPSS 22.0 software (Chicago, USA) was utilized for the data analysis. The images were created with Graph Pad Prism 8.0 (San Diego, CA, USA) software. Data were presented as mean ± standard deviation. One-way ANOVA was used to compare differences between groups. Statistical difference was considered in cases of a *P* value of less than 0.05.

## Results

### SG improved the metabolic parameters and glucose homeostasis in DM rats with DKD

**Fig. 1:**
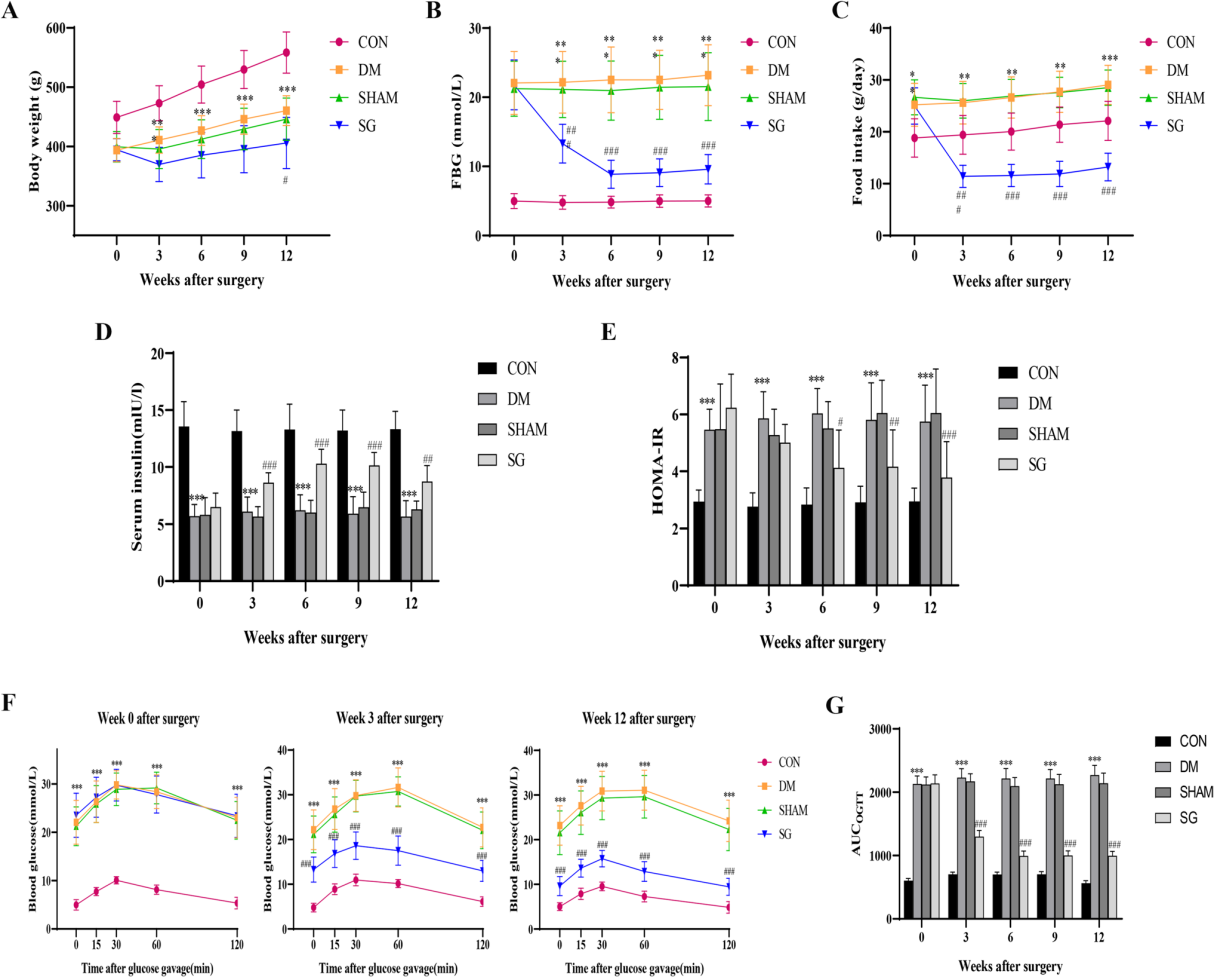
The advantageous effects of SG on glucose metabolism in DKD rats. **A**–**C** Body weight, FBG, and food intake showed significant improvement in the SG group compared with those of SHAM group. **D**, **E** SG triggered significant increase of serum insulin and decrease of HOMA-IR. **F** Blood glucose level at week 0, 3, and 12, respectively. **G** AUC of OGTT in each group. **P < 0.01, ***P < 0.001 versus control group; #*P* < 0.05, ##*P* < 0.01, ###*P* < 0.001 versus SHAM group

Compared with the control group, rats in DM group showed typical diabetic symptoms including decreased body weight (Fig. 1A), increased blood glucose and food intake (Fig. 1B, C), and decreased serum insulin and insulin sensitivity (Fig. 1D, E). These indices were reversed after SG compared with SHAM group. OGTT experiment revealed that compared with SHAM group, the IR was significantly alleviated in SG group at postoperative week 3 and lasted until week 12 (Fig. 1F, G). This suggested that SG could improve the metabolic parameters and glucose homeostasis in DM rats.

### SG ameliorated renal function in DM rats with DKD

**Fig. 2:**
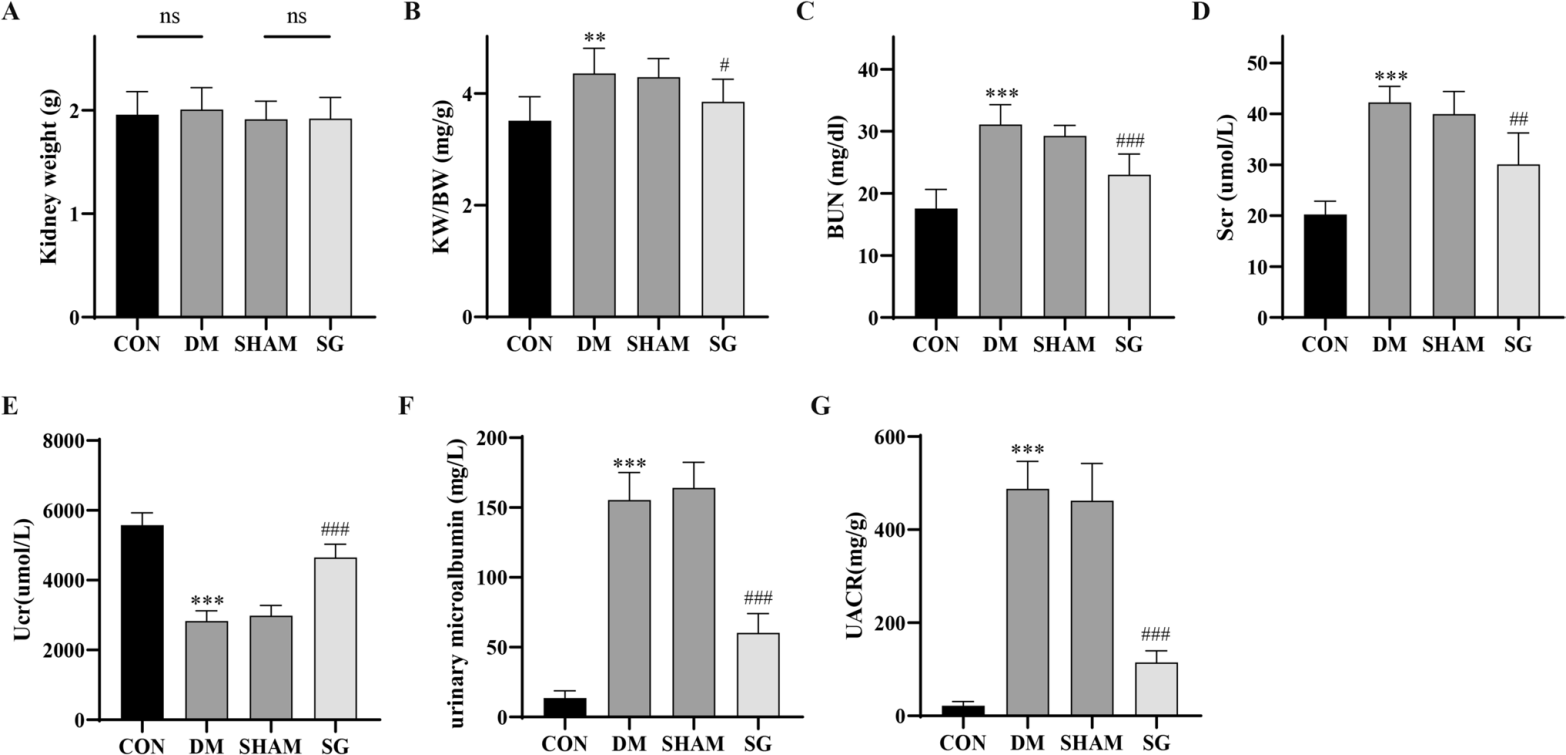
Comparison of kidney weight (**A**), KW/BW (**B**), BUN (**C**), Scr (**D**), Ucr (**E**), urinary microalbumin (**F**), and UACR (**G**) in each group; ns, no significance; *P < 0.05, **P < 0.01, ***P < 0.001 versus control group; #*P* < 0.05, ##*P* < 0.01, ###*P* < 0.001 versus SHAM group

There were no statistical differences in the kidney weights between control and DM group, and between SHAM group and SG group, respectively (*P* > 0.05, Fig. 2A). However, the kidney weight-to-body weight ratio (KW/BW ratio) was significantly increased in DM group compared with that of control. SG could dramatically decrease KW/BW ratio compared with that of SHAM group (Fig. 2B). Compared with control group, there was significant increase in the serum BUN and serum Cr (Scr) in DM group. Their concentrations showed significant decrease after SG compared with SHAM group (Fig. 2C, D). Besides, there was obvious increase in urinary creatinine (Ucr) and significant decrease in urinary microalbumin of the SG group compared with these of SHAM group (Fig. 2E, F). Consistently, SG could significantly reduce the UACR serving as a sensitive marker for renal function (Fig. 2G). These data indicated that SG could attenuate renal function in DM rats with DKD.

### SG attenuated the histological and morphological structures in DM rats with DKD

**Fig. 3:**
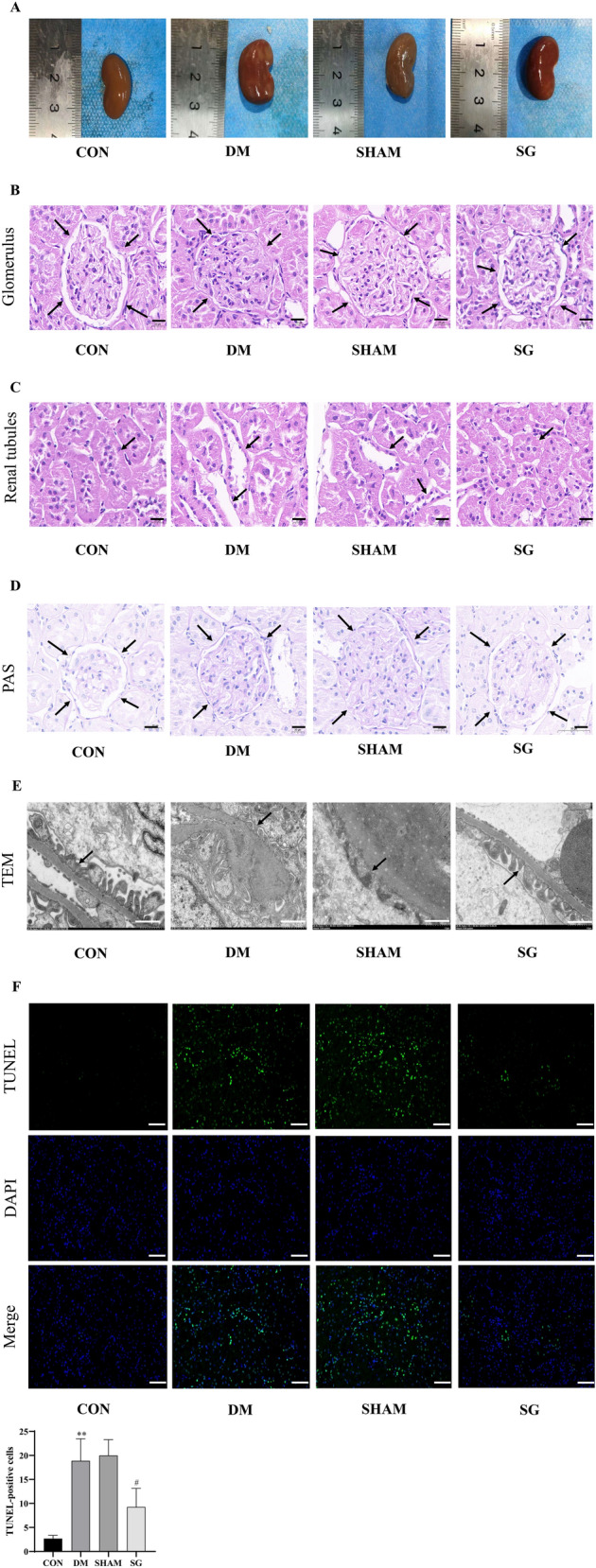
SG attenuated the histological and morphological injuries in DKD rats, which was associated with the alleviating effects on the injury of renal tubules. **A** Renal morphology. **B**, **C** HE staining for glomeruli and renal tubules. The scale bar represented 20 μm. **D** PAS staining for renal cortex. The scale bar represented 20 μm. **E** TEM findings. The scale bar represented 1 μm. **F** TUNEL assay indicated significant decrease of renal tubular injury after SG compared with SHAM group. The scale bar represented 100 μm. Arrows, the site with representative features. **P < 0.01 versus control group; #*P* < 0.05 versus SHAM group

Significant increase was seen in the number of brown fibrous lesions in kidneys representing more renal injuries in the DM group compared with control group, while the SG group showed significant reduction in the area of brown fibrosis compared with the SHAM group. Meanwhile, SG could attenuate the renal injuries featured by presence of more sites that were ruddy and smooth (Fig. 3A). In the SG group, we observed the reversion of the typical pathological features of DKD included proliferation of mesangial cells, thickening of the GBM, and dilatation of the lumen of the renal tubules with reduced or even absent microvilli by HE staining and PAS staining (Fig. 3B–D). TEM indicated that the GBM thickness was considerably lower, and podocyte foot process was wider, together with increased number of podocytes in SG group compared with that of SHAM group (Fig. 3E). TUNEL assay was performed to find that SG significantly alleviated cell death in renal tubules, especially the proximal tubules (Fig. 3F). Taken together, SG could attenuate the histological and morphological injuries in DKD rats, which may be associated with the alleviating effects on renal tubular injuries.

### SG down-regulated ferroptosis in renal tubules in DM rats with DKD

**Fig. 4:**
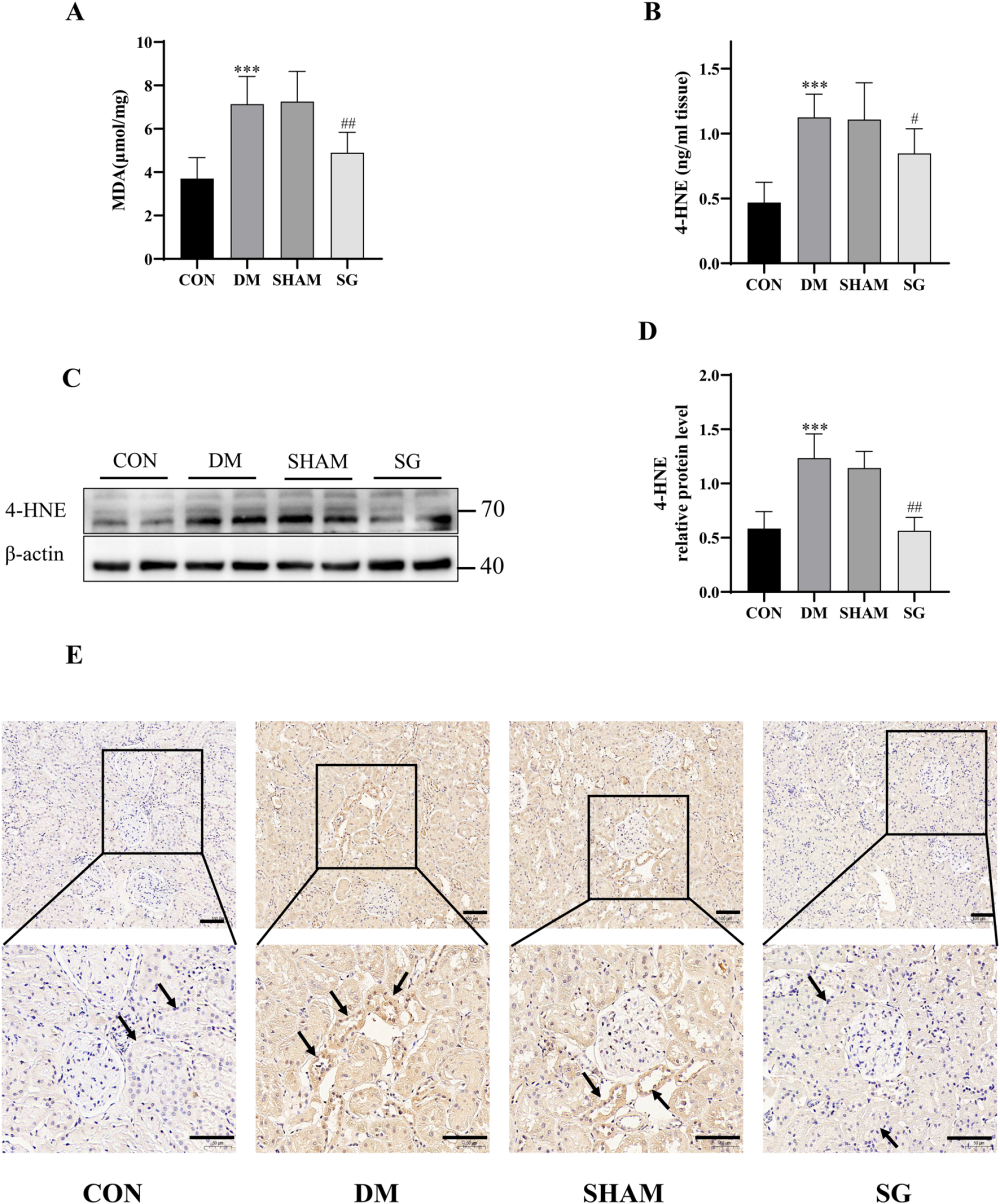
SG could alleviate the renal tubular ferroptosis in rats with DKD. **A**–**D** ELISA and Western blot analysis indicated that both MDA and 4-HNE showed significant decrease in SG group compared with SHAM group. **E** IHC showed that ferroptosis occurred mainly in the renal tubules rather than glomeruli. The scale bar represented 100 μm. Arrows, the site with representative features. ***P < 0.001 versus control group; #*P* < 0.05 versus SHAM group; ##*P* < 0.01 versus SHAM group

The majority of studies on the tubular injuries in DKD had focused on cellular apoptosis [20]. Bariatric surgery had been reported to significantly alleviate apoptosis and oxidative stress in the DKD [7]. However, cell death is complex, and bariatric surgery, especially SG, may ameliorate renal lesion through other types of death. Recently, renal tubular ferroptosis, a new type of cell death, has been reported to promote the progression of DKD [11]. Therefore, we tested whether SG could attenuate the renal tubular ferroptosis. We determined the concentrations of MDA and 4-HNE in tissue homogenate of renal cortexes that served as important markers of ferroptosis, as MDA and 4-HNE were products of lipid peroxidation that were mainly located inside the cells. ELISA and Western blot analysis indicated that both MDA and 4-HNE showed significant reduction in SG group compared with those of the SHAM group (Fig. 4A–D). IHC for 4-HNE showed that ferroptosis occurred mainly in the renal tubules rather than glomeruli, and SG could significantly repress the level of ferroptosis in renal tubules (Fig. 4E). This suggested that SG could alleviate the ferroptosis in renal tubules in DM rats with DKD.

### Effects of SG on ferroptosis-associated regulatory factors in DM rats with DKD

**Fig. 5:**
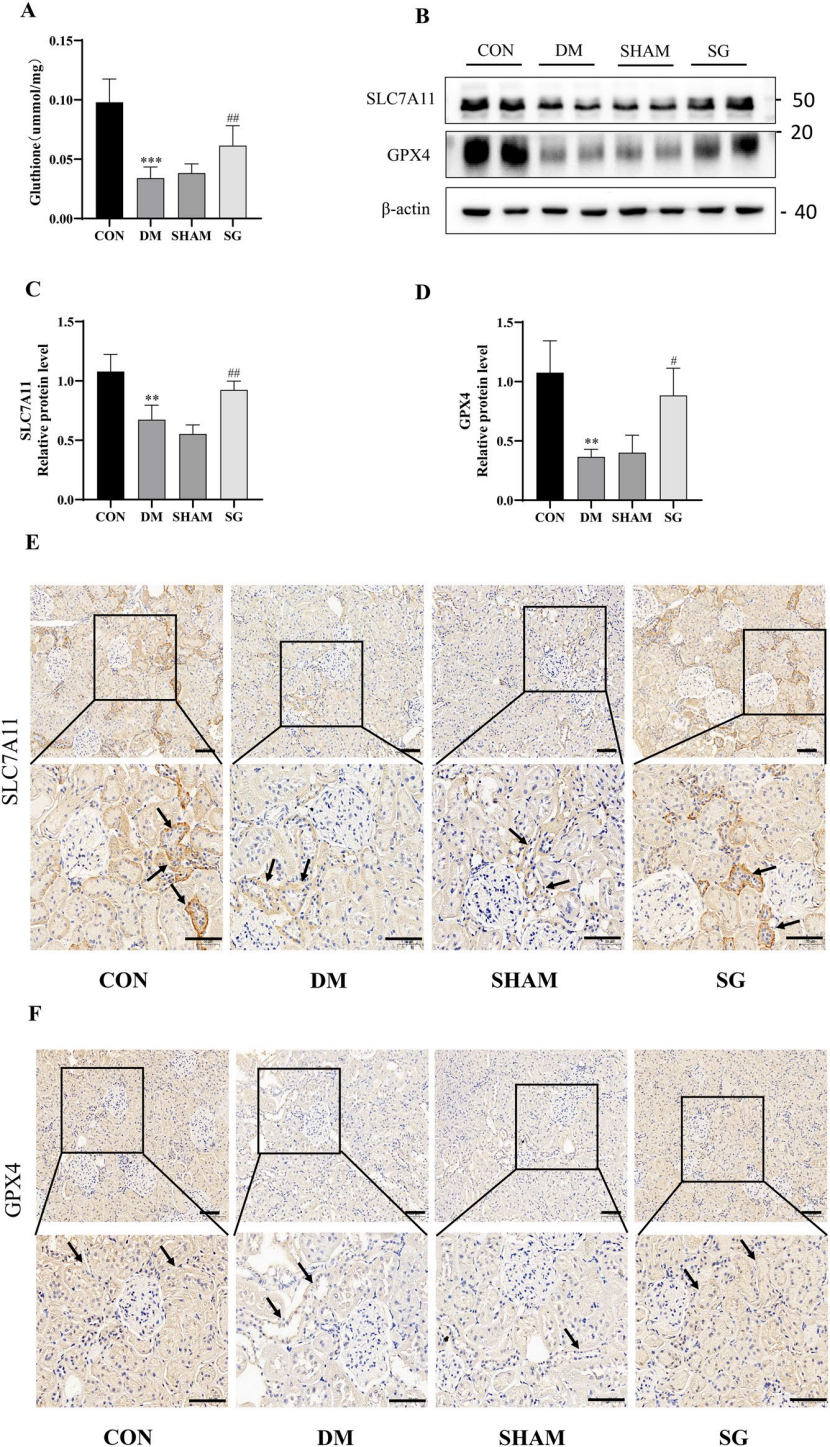
SG exerted anti-ferroptosis function through the SLC7A11–GSH–GPX4 axis. **A** SG significantly increased the GSH level compared with the SHAM group. **B**–**D** SG induced significant increase in SLC7A11 and GPX4 compared with those of the SHAM group. **E**, **F** SLC7A11 and GPX4 were mainly expressed in the tubules rather than the glomeruli, which showed remarkable increase in the renal tubules after SG. The scale bar represented 100 μm. Arrows, the site with representative features. **P < 0.01, ***P < 0.001 versus control group; #*P* < 0.05, ##*P* < 0.01 versus SHAM group

To investigate how SG modulated the ferroptosis, we then determined the expression of SLC3A2, SLC7A11, and GPX4 and the concentration of GSH, respectively. Our data showed that GSH level showed significant increase in SG group compared with that of SHAM group (Fig. 5A). Meanwhile, western blot analysis indicated significant increase of SLC7A11 and GPX4 in SG group compared with those in the SHAM group (Fig. 5B–D). However, there was no significant alteration in SLC3A2 between SG and SHAM group (Supplementary Fig. 2). In addition, IHC showed that SLC7A11 and GPX4 were mainly expressed in the renal tubules (Fig. 5E, F), rather than the glomeruli. Moreover, the expression of GPX4 and SLC7A11 showed remarkable increase in the renal tubules after SG. These indicated that SLC7A11-GSH-GPX4 axis played a crucial role in the anti-ferroptosis effects induced by SG.

### High glucose stimulated renal tubular TGF-β1 secretion that activated ferroptosis through the TGF-β1/Smad3 signaling pathway in the HK-2 cells

**Fig. 6:**
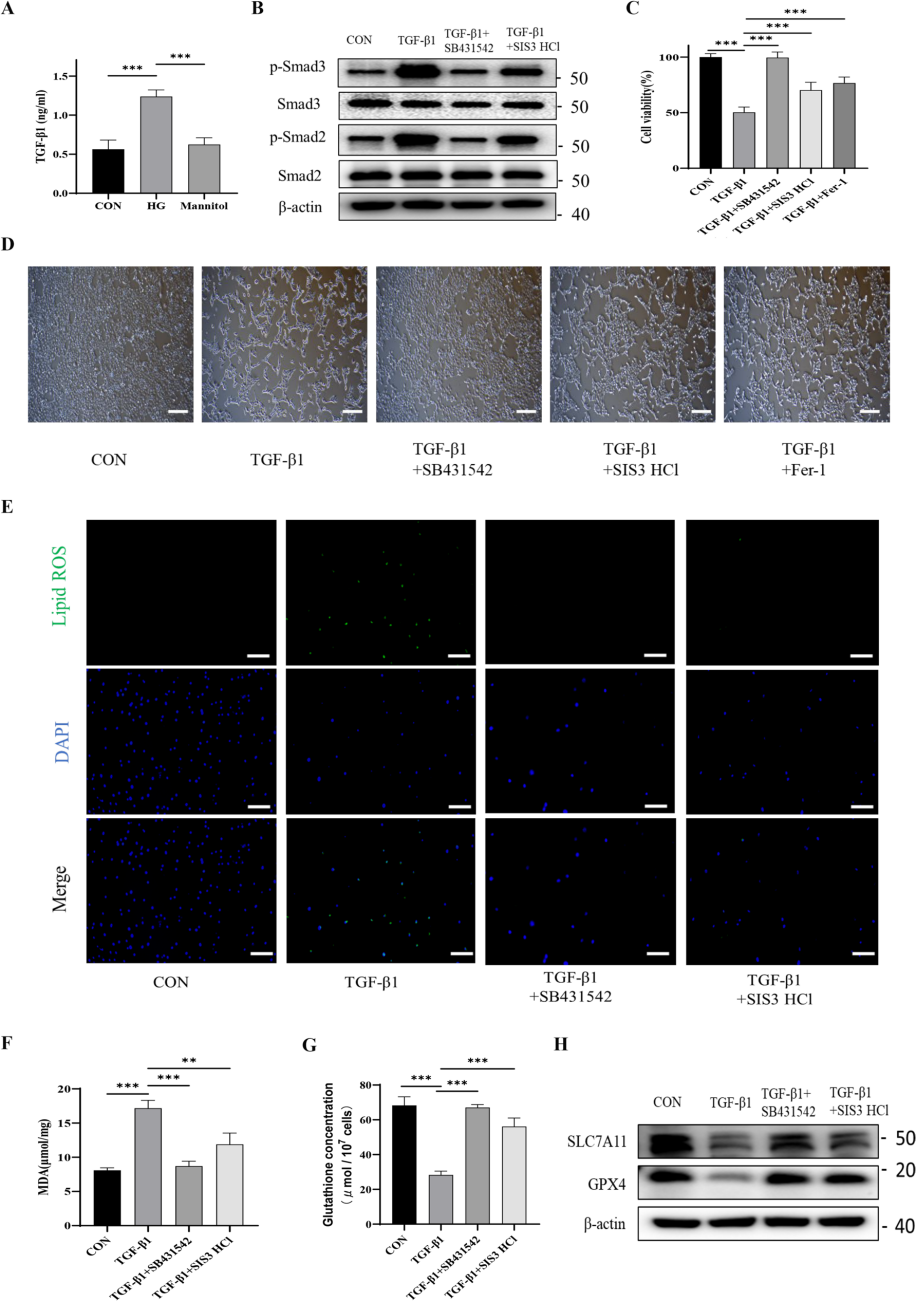
High glucose stimulated renal tubular TGF-β1 secretion, which then activated ferroptosis through the TGF-β1/Smad3 signaling pathway in the HK-2 cell line. **A** HG stimulated the release of TGF-β1 from HK-2 cells as compared to the control. **B** TGF-β1 induced upregulation of p-Smad3 and p-Smad2. **C**, **D** TGF-β1-induced ferroptosis was significantly reversed by SB431542, SIS3 HCl, and Fer-1. **E**, **F** Lipid-ROS and MDA assay confirmed that TGF-β1 could trigger the significant increase of ferroptosis. **G**, **H** TGF-β1 significantly induced the down-regulation of GSH, SLC7A11, and GPX4, respectively, which was obviously reversed in the presence of TGF-β1/Smad3 inhibitors. The scale bar represented 100 μm. **P < 0.01, ***P < 0.001

Recently, it has been reported that high glucose (HG) can promote ferroptosis in renal tubules [18]. Meanwhile, TGF-β1 is significantly elevated in DKD patients, and a strong correlation with the progression of DKD has been demonstrated in some studies [21, 22]. To further elaborate whether SG could exert its function of repressing renal tubular ferroptosis through its anti-hyperglycemia effect, we then performed cellular experiments using HK-2 cells, a proximal tubular epithelial cell line. We first used ELISA to determine TGF-β1 concentration in HK-2 cells cultured in conditioned medium supplemented with glucose and mannitol. The latter could increase osmotic pressure without being absorbed by cells, which excluded the effects on the changes of glucose-induced osmotic pressure. Our data indicated that HG stimulated the release of TGF-β1 in renal proximal tubular epithelial cells (Fig. 6A). As the TGF-β1/Smad signaling pathway was a canonical pathway associated with TGF-β1, we investigated the roles of TGF-β1/Smad3 on renal tubular ferroptosis in the presence of TGF-β1 receptor inhibitor (i.e., SB431542) and Smad3 phosphorylation inhibitor (i.e., SIS3 HCl), respectively (Fig. 6B). Cellular viability test indicated that TGF-β1 could significantly induce HK-2 cells ferroptosis, which was also significantly reversed by SB431542, SIS3 HCl, and Fer-1 (a ferroptosis inhibitor, severed as the positive control) (Fig. 6C, D). In addition, we determined the intracellular levels of lipid-ROS and MDA, which showed that TGF-β1 could trigger the significant increase of ferroptosis (Fig. 6E, F). Interestingly, SB431542 and SIS3 HCl can effectively reverse the effect induced by TGF-β1, suggesting that TGF-β1 promoted ferroptosis in renal tubular cells via the TGF-β1/Smad3 signaling pathway. Finally, we probed glutathione, SLC3A2, SLC7A11, and GPX4 serving as classical anti-ferroptosis regulatory molecules, which demonstrated that TGF-β1 significantly induced the down-regulation of GSH, SLC7A11, and GPX4, respectively. Such phenomenon was obviously reversed in the presence of TGF-β1/Smad3 inhibitors (Fig. 6G, H). In addition, we found no significant change in SLC3A2 (Supplementary Fig. 3). All these data implied that the activation of TGF-β1/Smad3 signaling pathway induced by high glucose was closely related to the ferroptosis in renal tubules.

### SG remarkably down-regulated the TGF-β1/Smad3 signaling pathway in DM rats with DKD

**Fig. 7:**
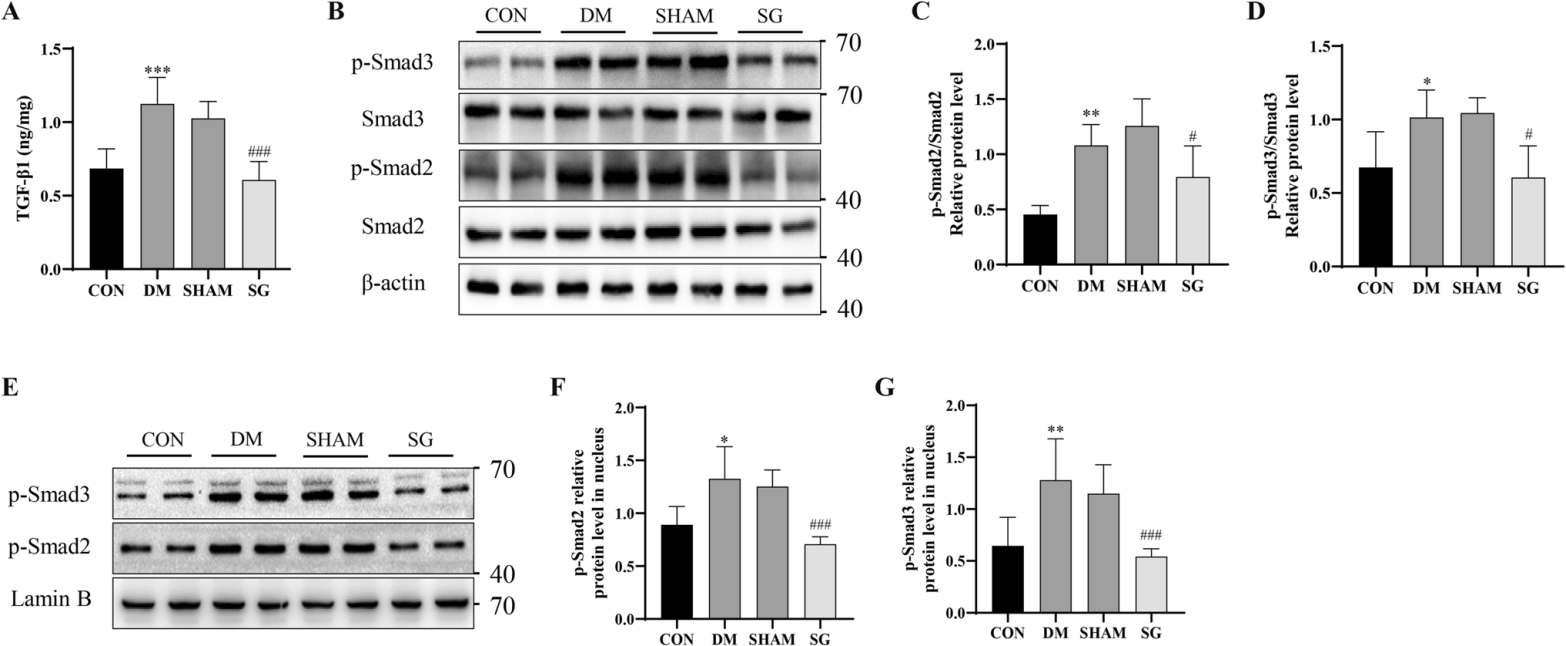
SG remarkably down-regulated the TGF-β1/Smad3 signaling pathway in DM rats with DKD. **A** TGF-β1 expression was significantly down-regulated after SG compared with SHAM group. **B**–**D** The p-Smad2/Smad2 and p-Smad3/Smad3 expression was significantly decreased in the SG group compared with those of SHAM group. **E**–**G** SG somehow reduced their nuclear localization of p-Smad2/3 compared to the SHAM group. *P < 0.05, **P < 0.01 versus control group; #*P* < 0.05, ###*P* < 0.001 versus SHAM group

To explore whether SG could suppress the TGF-β1/Smad3 signaling pathway that was proved to promote renal tubular ferroptosis, we examined the relevant indicators in vivo. The concentration of TGF-β1 in kidney cortexes was up-regulated in DM group compared with that of control, while its concentration was significantly down-regulated after SG compared with SHAM group using ESLIA(Fig. 7A). Similarly, the expression of p-Smad2/Smad2 and p-Smad3/Smad3 was significantly down-regulated in the SG group compared with those of SHAM group (Fig. 7B–D). Since p-Smad2/3 exerted their effects through entering the nucleus [23], we further examined the nuclear expression of these two molecules, which showed that p-Smad2/3 nucleus expression was elevated in DM group, while the SG reduced their nuclear localization compared to the SHAM group (Fig. 7E–G).

## Discussion

Bariatric surgery has been considered as a potential alternative for treating DKD [24]. Among the weight-loss strategies available, SG has been considered as an effective and sustained option. However, our understanding on the SG for treating DKD is still limited due to a lack of high-quality studies [25]. In this study, we established a DM rat model complicated with DKD, and investigated the effects of SG on such condition. Our data showed that SG could attenuate the DKD symptoms by reducing ferroptosis in renal tubules, and the inhibition of TGF-β1/Smad signaling pathway in the presence of anti-hyperglycemic effects induced by SG was crucial for this process.

SG has been reported to attenuate the related symptoms in DKD patients such as proteinuria [26]. For DKD patients showed poor response to conventional therapy, SG had been considered a promising therapy [24]. In this study, we proved that SG could attenuate the DKD symptoms in DM rats. Our data showed that SG resulted in a decrease in KW/BW serving as an effective predictor for DKD. Meanwhile, based on concentrations of Cr and urea nitrogen in serum and urine, our data showed that SG could substantially mend the renal function in DKD rats. The typical features of DKD included histological alterations in renal tissues, such as glomerular mesangial hyperplasia, accumulation of excess extracellular matrix proteins, reduction in the brush border of renal tubular epithelial cells, and dilatation of the luminal compartment [12]. Moreover, we confirmed that SG could significantly improve the histological and morphological changes of glomeruli and tubules in diabetic nephropathy according to the HE and PAS staining, as well as TEM findings. TUNEL assay was performed to determine the cellular death to confirm the site of tissue injury. Interestingly, we found that cellular death in DM rats with DKD mainly occurred in the renal tubules rather than glomeruli. Besides, SG could attenuate the injury of the tubular epithelial cells. On this basis, we found that the attenuation of SG on DKD may be related to its protective effect on renal tubular injury.

Inhibition of the renin–angiotensin system (RAS) with ACEI/ARB, mainly targeting the glomerulus, is currently the main treatment option for DKD [27]. Indeed, it is beneficial in controlling the DKD progression; however, it is not effective in reversing the conditions. Thus, there might be other mechanisms responsible for the onset of DKD. Recently, increasing studies have focused on the roles of renal tubules in DKD [28], among which SGLT2 blockers targeted the renal tubules have been used with significant clinical benefits [29, 30]. Therefore, some researchers propose that renal tubules play a central role in the progression of DKD, which is called the tubular hypothesis [1]. Renal tubular epithelial injury was closely related to the renal injury, and the programmed cell death was crucial in this process [11]. To date, the major types of death in the tubular epithelial cells of DKD included apoptosis, necroptosis, and pyroptosis [31–33]. Serving as a new type of cellular death [34], ferroptosis has been recently reported in tubular injury in DKD [11]. MDA and 4-HNE, as lipid peroxidation products tend to bind with proteins and DNA, were effective ferroptosis-related indicators that were closely related to ferroptosis [35]. In our study, SG effectively lowered the level of ferroptosis in DM rats with DKD, featuring by significant decrease of MDA and 4-HNE compared with the SHAM group. Additionally, we found that ferroptosis in DKD occurred mainly in the renal tubules rather than glomeruli, and the major site with significant decrease of ferroptosis after SG was also located in the renal tubules by IHC. These indicated that SG could inhibit ferroptosis in renal tubules. As a pivotal inhibitory pathway of ferroptosis, SLC7A11–GSH–GPX4 axis has attracted enormous attention and been targeted for treating ferroptosis-related diseases [36]. In our study, SG markedly up-regulated the level of GSH and the expression of SLC7A11 and GPX4 in renal tubular of DM rats with DKD. Therefore, SG could inhibit the renal tubular ferroptosis in DKD rats by activating the SLC7A11–GSH–GPX4 axis.

TGF-β1 expressed by kidney cells is a pleiotropic cytokine involved in the angiogenesis and immunomodulation. Overactivation of TGF-β signaling pathway has been implicated as a critical factor in DKD progression [37]. TGF-β1 signaling pathway is divided into canonical and non-canonical pathways, respectively. The canonical pathway was activated through phosphorylation activation of Smad2/3. In the non-canonical pathway, TGF-β1 activated the Smad-independent pathways, such as Ras/Raf/MEK, PI3K/Akt/mTOR, MAPK/JNK, and RhoA/RocK pathways. In line with the previous studies [38, 39], our data showed that HG was capable of inducing release of TGF-β1 from HK-2 cells. Besides, TGF-β1 triggered the ferroptosis in renal tubular epithelial cells, which was visibly reversed by inhibiting the TGF-β1 receptor and phosphorylation of Smad3. Meanwhile, TGF-β1 significantly down-regulated SLC7A11–GSH–GPX4 axis, which was abrogated in the presence of inhibition of TGF-β1 receptor and p-Smad3. On this basis, TGF-β1 showed pro-ferroptosis effects through Smad3 phosphorylation. Interestingly, in vivo experiments confirmed that SG could remarkably reduce the concentration of TGF-β1 in kidneys in the circumstance of DKD, which may be related to its anti-hyperglycemic effects. Meanwhile, we observed that SG effectively suppressed the TGF-β1/Smad3 signaling pathway, which demonstrated that SG inhibited renal tubular ferroptosis through down-regulating the TGF-β1/Smad3 signaling pathway.

There are certain limitations in our study. First, we used HK-2 cell line instead of primary renal tubular epithelial cells to verify the relationship between HG, TGF-β1/Smad3 signaling pathway and ferroptosis due to technical limitation. Second, rescue experiments were not performed in animals due to limited experimental conditions. Finally, as mentioned above, we were not able to investigate the roles of non-classical pathways of TGF-β1 in renal tubular ferroptosis.

In DM rats with DKD, SG led to decrease of TGF-β1 and inhibited the activation of the TGF-β1/Smad3 signaling pathway by reversing the hyperglycemia. Then, the SLC7A11–GSH–GPX4 axis was up-regulated, thereby inhibiting the ferroptosis in the renal tubules. Our study provided a promising perspective for the treatment of DKD.

### Supplementary Information

Below is the link to the electronic supplementary material.

Supplementary file1 (PDF 320 KB)

## Data Availability

The detailed data used to support the findings of this study are available from the corresponding author upon request.
