# Supplementary material for: Sleeve gastrectomy links the attenuation of diabetic kidney disease to the inhibition of renal tubular ferroptosis through down-regulating TGF-β1/Smad3 signaling pathway
Source: J Endocrinol Invest. 2024 Mar 21;47(7):1763–76. doi: 10.1007/s40618-023-02267-1 (PMC11196306; doi:10.1007/s40618-023-02267-1)
Supplement: Supplementary file 1 — Supplementary file1 (PDF 320 KB) [file 40618_2023_2267_MOESM1_ESM.pdf]

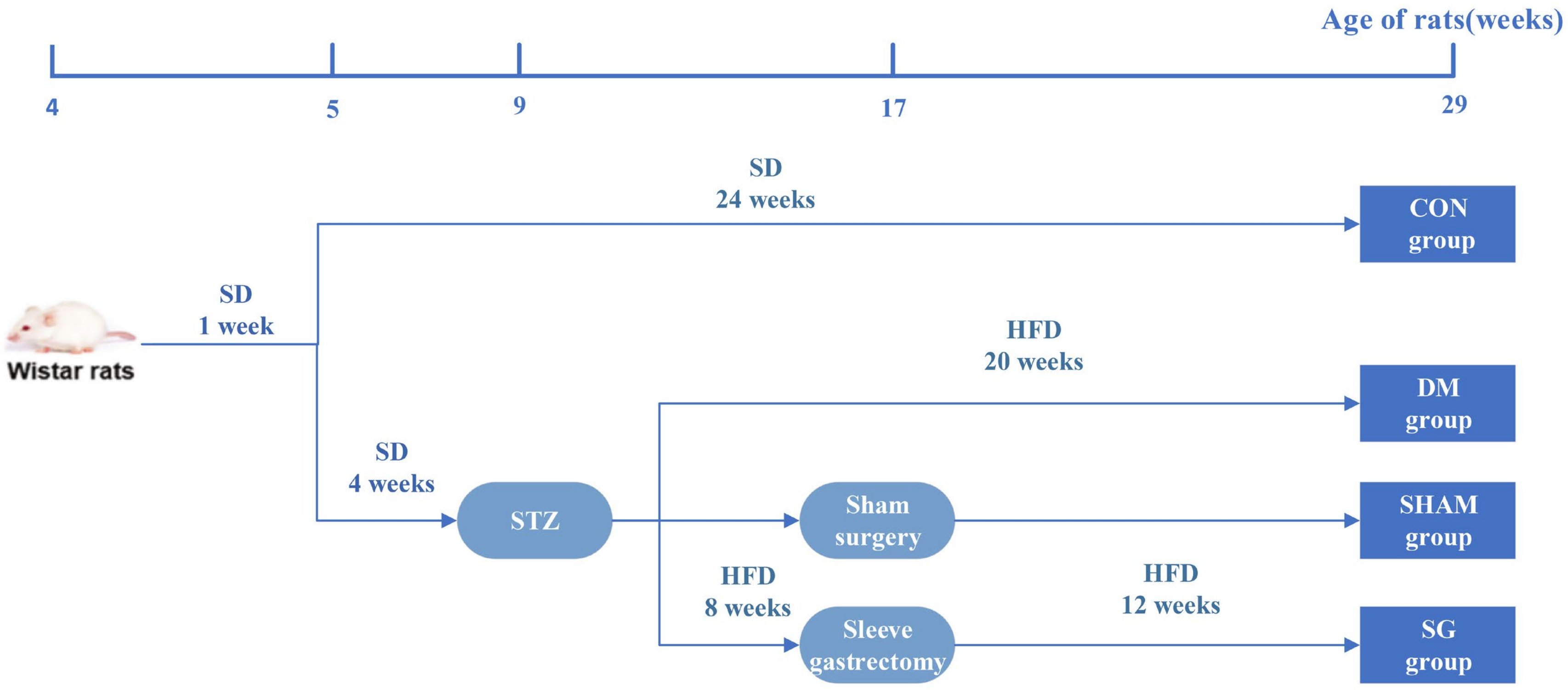

Supplementary figure 1: Schematic description of the experimental design used to establish the animal model.

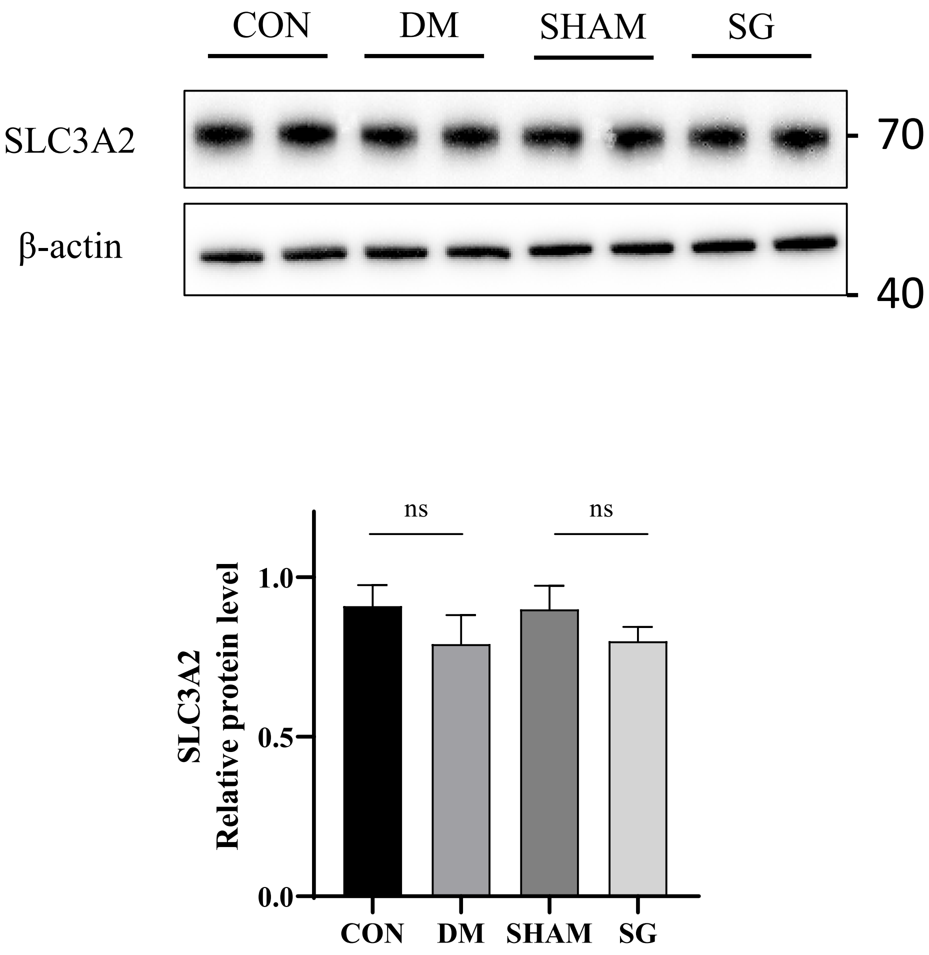

Supplementary figure 2: Western blot analysis of SLC3A2 protein expression in each group.

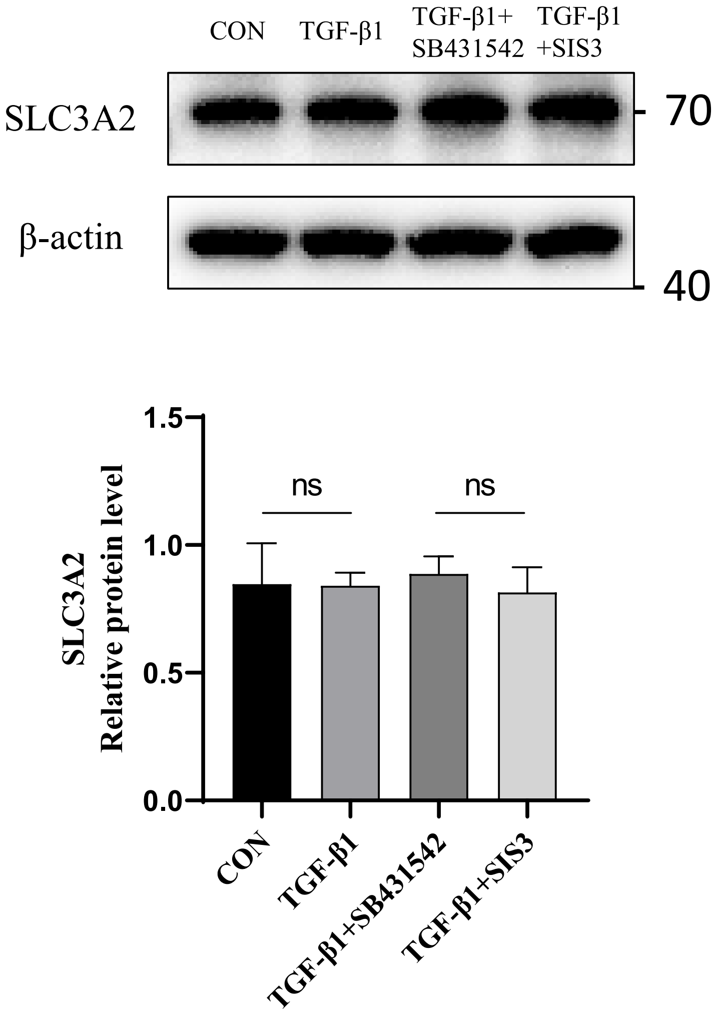

Supplementary figure 3: Western blot analysis of SLC3A2 protein expression in HK-2 under indicated treatment.
